# Supplementary material for: In-utero cell transplantation for hypophosphatasia with gene-edited hESC-derived MSCs in a murine model
Source: Mol Ther Adv. 2026 Feb 13;34(2):201696. doi: 10.1016/j.omta.2026.201696 (PMC13148942; doi:10.1016/j.omta.2026.201696)
Supplement: Document S1. Figures S1–S3 [file mmc1.pdf]

OMTA, Volume *34*

Supplemental information

In-utero cell transplantation for hypophosphatasia with gene-edited hESC-derived MSCs in a murine model

Naoya Kitamura, Akihiro Hasegawa, Aki Takahashi-Nakamura, Masataka Kasahara, Hidenori Akutsu, Haruhiko Sago, Osamu Samura, Aikou Okamoto, and Akihiro Umezawa

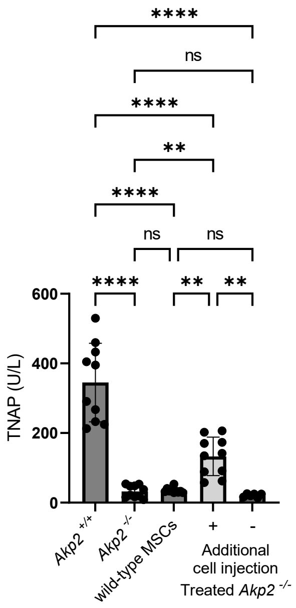

Figure S1. Serum TNAP levels at 14 days postnatally.

Akp2 $^{+/+}$: Wild-type *Akp2* mice. *Akp2* $^{-/-}$: Untreated HPP mice. Wild-type MSCs: Mice administered MSCs without gene transfer at E14.5 and postnatal day 8. Additional cell injection Treated *Akp2* $^{-/-}$: Groups receiving (+) or not receiving (–) an additional injection at postnatal day 8 after receiving TNAP-overexpressing human ESC-derived MSCs at E14.5.

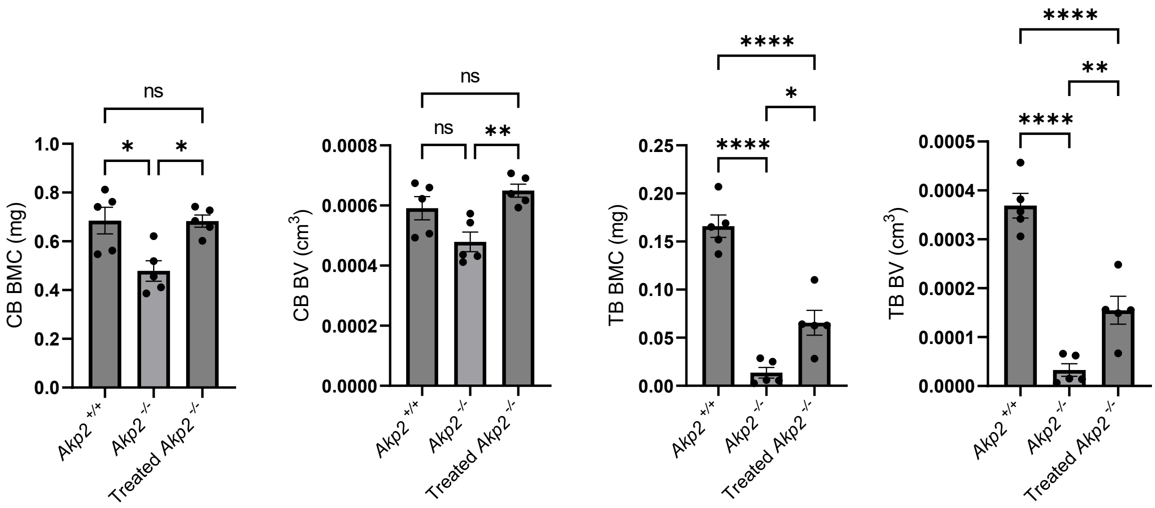

Figure S2. Additional micro-CT analysis data of bone formation 14 days postnatally.
BMC represents the total calcification level recognized as bone within the ROI, and BV indicates bone volume. Data are shown for cortical (CB) and trabecular (TB) bone, respectively. The treatment resulted in a significant improvement in both parameters compared with the untreated *Akp2* $^{-/-}$ group.

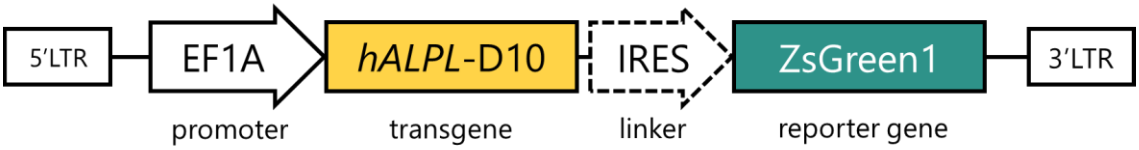

Figure S3. Schematic map of the lentiviral expression vector used to transduce MSCs with the hALPL-D10 and ZsGreen genes.
The two genes are co-expressed from a single bicistronic transcript via an IRES sequence.
