# Supplementary material for: In-utero cell transplantation for hypophosphatasia with gene-edited hESC-derived MSCs in a murine model
Source: Mol Ther Adv. 2026 Feb 13;34(2):201696. doi: 10.1016/j.omta.2026.201696 (PMC13148942; doi:10.1016/j.omta.2026.201696)
Supplement: Document S2. Article plus supplemental information [file mmc2.pdf]

Molecular Therapy
Advances
Original Article

In-utero cell transplantation for hypophosphatasia with gene-edited hESC-derived MSCs in a murine model

Naoya Kitamura,[1,2,6] Akihiro Hasegawa,[1,2,6] Aki Takahashi-Nakamura,[3] Masataka Kasahara,[3] Hidenori Akutsu,[1] Haruhiko Sago,[4,5] Osamu Samura,[2] Aikou Okamoto,[2] and Akihiro Umezawa[1]

[1]Center for Regenerative Medicine, National Center for Child Health and Development, Tokyo 157-8535, Japan; [2]Department of Obstetrics and Gynecology, The Jikei University School of Medicine, Tokyo 105-8461, Japan; [3]Department of Pharmacology, Tokyo Dental College, Tokyo 101-0061, Japan; [4]International University of Health and Welfare, Chiba 286-8520, Japan; [5]Sanno Birth Center, Tokyo 107-0052, Japan

The perinatal severe form of hypophosphatasia (HPP), caused by a deficiency in tissue-nonspecific alkaline phosphatase (TNAP), which is involved in bone metabolism, is a disease that manifests during fetal development. However, no established treatment can be performed in utero, and invasive treatment must be continued for a lifetime immediately after birth due to irreversible thoracic hypoplasia. We have developed an *ex vivo* in-utero gene cell therapy as a new treatment that can intervene at the fetal stage. We transduced the *hALPL-D10* gene into human embryonic stem cell-derived mesenchymal stem cells to generate cells that overexpress TNAP. We transplanted the cells into the fetuses of HPP mice without immunosuppression by total body irradiation. The primary strength of this methodology lies in the fact that all experiments were conducted using immunocompetent animals with human cells. Compared to the untreated group, the treated group showed an extended survival rate (p = 0.005). In addition, the treated group showed significant weight gain (day 0 [p = 0.024], day 7 [p < 0.001], and day 14 [p = 0.017]) and improvement in bone lesions. *Ex vivo* in-utero gene cell therapy may be a new option for treating patients with HPP during gestation.

INTRODUCTION

Hypophosphatasia (HPP) is a rare hereditary disease caused by a deficiency in tissue-nonspecific alkaline phosphatase (TNAP), which leads to inadequate bone calcification.[1] TNAP plays a crucial role in hydrolyzing inorganic pyrophosphate (PPi), a potent inhibitor of bone mineralization. In HPP, the accumulation of PPi disrupts normal bone mineralization.[2] HPP is classified into six types based on the timing of onset, with the perinatal severe form being the most serious. This form is characterized by bone calcification disorders that manifest during gestation, with an estimated incidence of 1 in 297,000 in Europe and 1 in 150,000 in Japan.[3–5] Notably, irreversible chest hypoplasia develops in utero, leading to respiratory failure and, if left untreated, fatality within a month.[6] The primary treatment for HPP is enzyme replacement therapy (ERT) with asfotase alfa. While ERT has increased survival rates, its therapeutic effect remains insufficient for treating the irreversible bone disorders that progress during pregnancy. While prenatal diagnostic tools have improved detection,[7,8] effective fetal therapies do not exist, and current postnatal ERT is often insufficient to reverse skeletal deformities that developed in utero.[9] Therefore, it is extremely important to develop treatments that can be administered early in pregnancy to mitigate disease progression.[6] In this study, we developed human embryonic stem cell (hESC)-derived mesenchymal stem cells (MSCs) that overexpress TNAP, aiming to minimize invasiveness to the fetus and enhance the efficiency of ERT. Human ESC-derived MSCs offer unlimited scalability and few variations between cells compared to adult tissue-derived MSCs while maintaining robust immunomodulatory properties. They facilitate precise genetic engineering, making them ideal platforms for regenerative therapies and *ex vivo* gene editing applications without total body irradiation. Transplantation of these cells in utero in mice demonstrated an improved prognosis. *Ex vivo* in-utero gene cell therapy shows promise for effectively treating congenital metabolic disorders during the fetal stage.

RESULTS

Generation of TNAP-overexpressing cells

Human ESCs were differentiated into MSCs using previously established protocols. The differentiated cells exhibited the capacity to form osteoblasts, chondroblasts, and adipoblasts (Figure 1A). Flow cytometric analysis revealed that the cells expressed the MSC surface markers CD105, CD73, and CD90, without expression of the hematopoietic markers CD45, CD34, CD14, CD19, and HLA-DR (Figure 1B). The cells were subsequently transduced with a lentiviral vector harboring the *hALPL-D10* gene. Following transduction, hALPL-D10 expression increased by more than 200,000-fold, and

Received 26 May 2025; accepted 9 February 2026;
https://doi.org/10.1016/j.omta.2026.201696.

[6]These authors contributed equally

Correspondence: Akihiro Umezawa, Center for Regenerative Medicine, National Center for Child Health and Development, Tokyo 157-8535, Japan.
E-mail: umezawa-a@ncchd.go.jp

Published by Elsevier Inc. on behalf of The American Society of Gene and Cell Therapy.
This is an open access article under the CC BY license (http://creativecommons.org/licenses/by/4.0/).

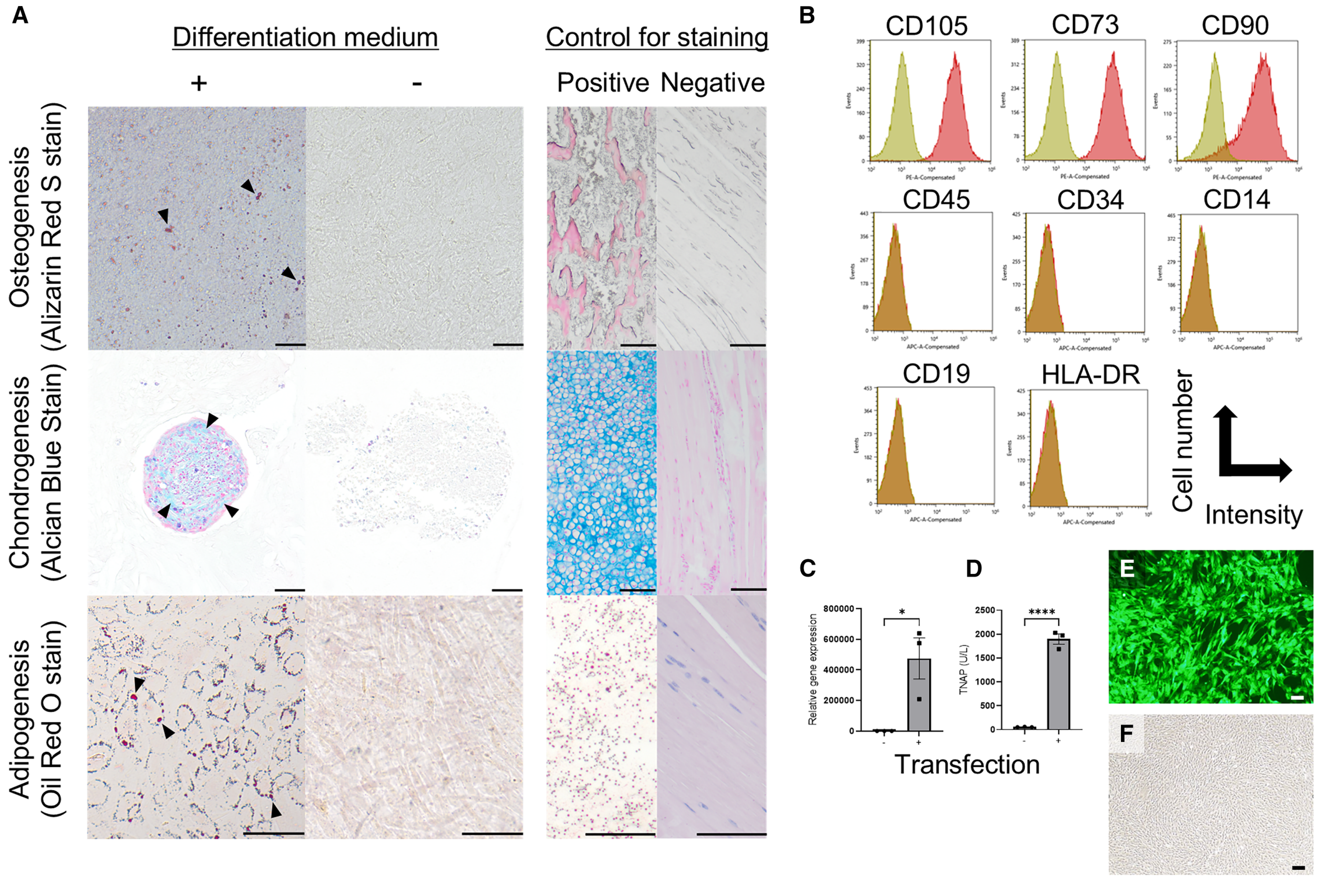

Figure 1. Cellular characterization of human ESC-derived cells as MSCs

(A) Pathology after 21 days of differentiation induction of human ESC-derived cells. Human ESC-derived cells differentiated into bone (Alizarin Red S stain), cartilage (Alcian blue stain), and fat (Oil Red O stain) using a differentiation medium. The black bar is 100 μm. The femur, xiphoid process, and fat around the femur of mice were selected as positive controls for staining, and the thigh muscle was chosen as a negative control.

(B) Flow cytometric analysis of phenotypic markers of human ESC-derived cells. ESC-derived cells were positive for CD105 (99.12%), CD73 (99.15%), and CD90 (98.96%) as makers for MSCs and negative for CD45 (0.95%), CD34 (0.89%), CD14 (0.88%), CD19 (0.72%), and HLA-DR (1.16%) as makers for hematopoietic stem cells ($n = 3$ in each group). The numbers in parentheses indicate the percentage of cells positive for each cell surface marker.

(C) The expression levels of the *hALPL-D10* gene in the transduced cells significantly increased ($p = 0.024$). Gene expression in the controls was regarded as equal to 1.0.

(D) TNAP in the supernatant of transduced cells (1898.35 ± 105.90 U/L) was significantly increased compared to that in non-transduced cells (56.18 ± 2.88 U/L; $p < 0.001$).

(E and F) Almost all human ESC-derived cells, after gene transfer, emitted fluorescence. The observation was conducted at ISO 800, with an exposure time of 555.6 ms. The white bar is 200 μm.

TNAP activity in the culture supernatant was markedly elevated compared with non-transduced cells (Figures 1C and 1D). Nearly all transduced cells exhibited ZsGreen fluorescence, confirming efficient gene delivery (Figures 1E and 1F).

TNAP-overexpressing human ESC-derived MSCs engrafted in the abdominal cavity of the fetus

Mating was carried out between *Akp2*$^{+/-}$ mice. On day 14.5 of gestation, 1.0×10^6 cells of TNAP-overexpressing human ESC-derived MSCs were administered intraperitoneally to the treated *Akp2*$^{-/-}$ group (Figure 2A). These cells were confirmed to be diffusely engrafted in the abdominal cavity of the fetuses on day 16.5 of gestation (Figure 2B). No cell transplantation was performed in the untreated *Akp2*$^{-/-}$ group throughout the observation period. Genotyping was performed after birth, and only *Akp2*$^{-/-}$ mice were selected for the treated and untreated *Akp2*$^{-/-}$ groups. *Akp2*$^{+/+}$ mice was selected as wild-type controls. The body weight at days 0, 7, and 14 after birth was lower in the untreated *Akp2*$^{-/-}$ group (day 0; 1.427 ± 0.035 g, day 7; 4.103 ± 0.160 g, and day 14; 7.180 ± 0.361 g) than in the *Akp2*$^{+/+}$ group (day 0; 1.562 ± 0.071 g, $p = 0.162$, day 7; 5.207 ± 0.295 g, $p = 0.004$, and day 14; 8.765 ± 0.492 g, $p = 0.064$). The treated *Akp2*$^{-/-}$ group (day 0; 1.633 ± 0.037 g, day 7; 5.719 ± 0.174 g, and day 14; 9.125 ± 0.551 g) showed a significant increase in body weight compared to the untreated *Akp2*$^{-/-}$ group (day 0; $p = 0.020$, day 7; $p < 0.001$, and day 14; $p = 0.020$). The treatment restored the body weight of *Akp2*$^{-/-}$ mice to a level similar to that of the *Akp2*$^{+/+}$ group

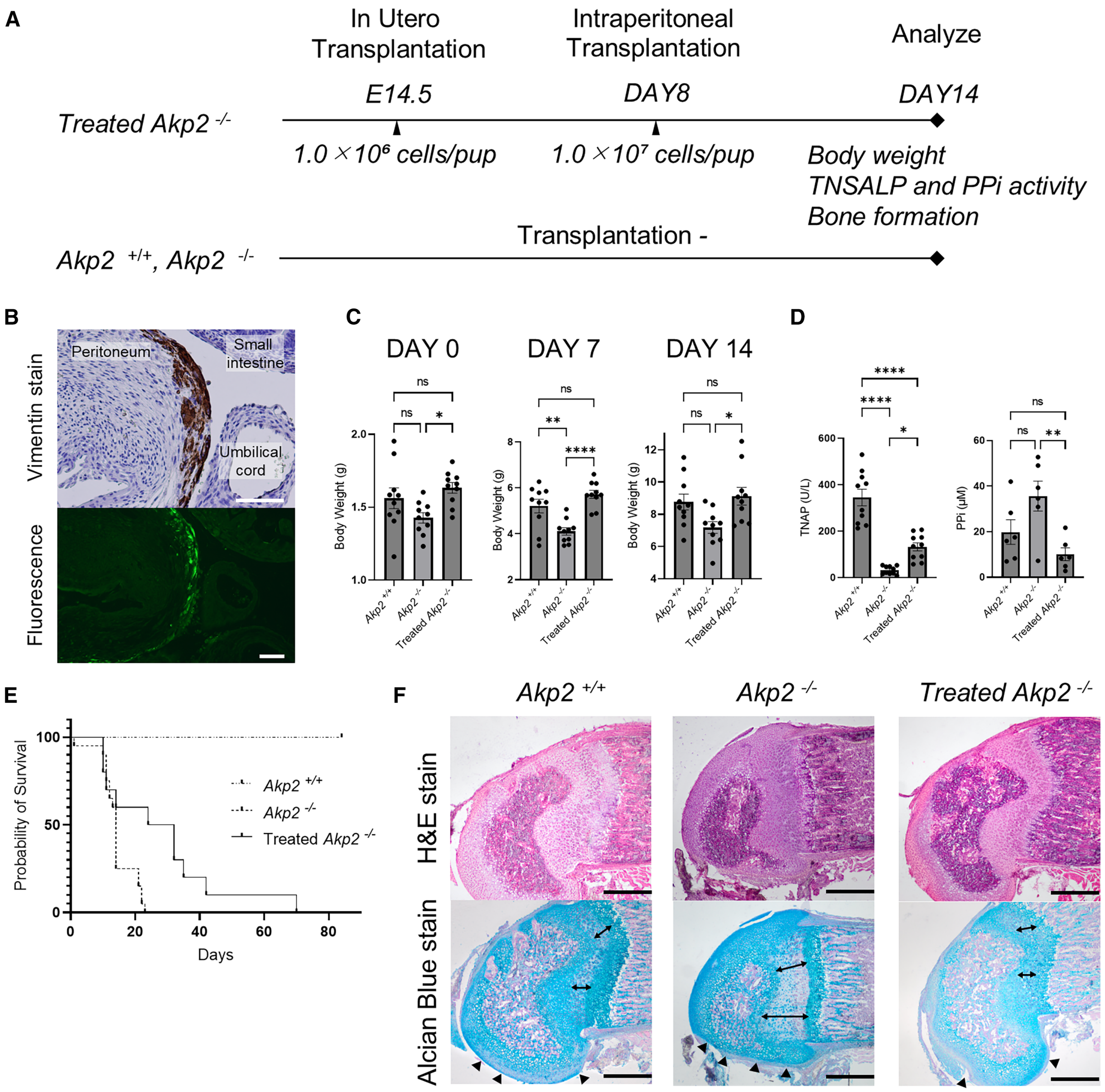

Figure 2. In-utero transplantation of TNAP-overexpressing human ESC-derived MSCs in *Akp2*$^{-/-}$ mice

(A) Protocol for cell transplantation in mice. In the treated group, cells were administered on gestation day 14.5 and postnatal day 8.

(B) Pathology of the fetal peritoneal cavity on the second day after fetal cell transplantation. Cells that were positive for human vimentin and emitted fluorescence were observed on the peritoneum on the wall. The white bar is 200 μm.

(C) Treatment increased the weight of the mice compared to the untreated group (day 0; $p = 0.020$, day 7; $p < 0.001$, and day 14; $p = 0.020$) ($n = 10$ in each group).

(D) Treatment significantly improved blood TNAP ($p < 0.001$, $n = 10$ in each group) and PPi ($p = 0.011$, $n = 6$ in each group) concentrations on postnatal day 14 compared to the untreated group.

(E) The survival rate after birth was significantly higher in the treated group than in the untreated group ($p = 0.005$).

(F) The distal femur was sectioned sagittally on postnatal day 14 and stained with H&E and Alcian blue. In the treated *Akp2*$^{-/-}$ group, cartilage formation in the growth plate was accelerated, and the shape of the femoral head became the same as that of the wild type. The black bar is 200 μm. Statistical significance is shown as *$p < 0.05$, **$p < 0.01$, and ****$p < 0.0001$. “ns” means “not significant.”

during all observation periods (Figure 2C). In the untreated *Akp2*$^{-/-}$ group, TNAP (32.22 ± 5.55 U/L) was significantly reduced, and PPi (35.62 ± 6.62 μM) accumulated compared to the *Akp2*$^{+/+}$ group (TNAP; 345.02 ± 35.68 U/L, $p < 0.001$ and PPi; 19.82 ± 5.41 μM, $p = 0.113$) due to the mutation in the *TNAP* gene. At 14 days of age, TNAP significantly increased and PPi significantly decreased in

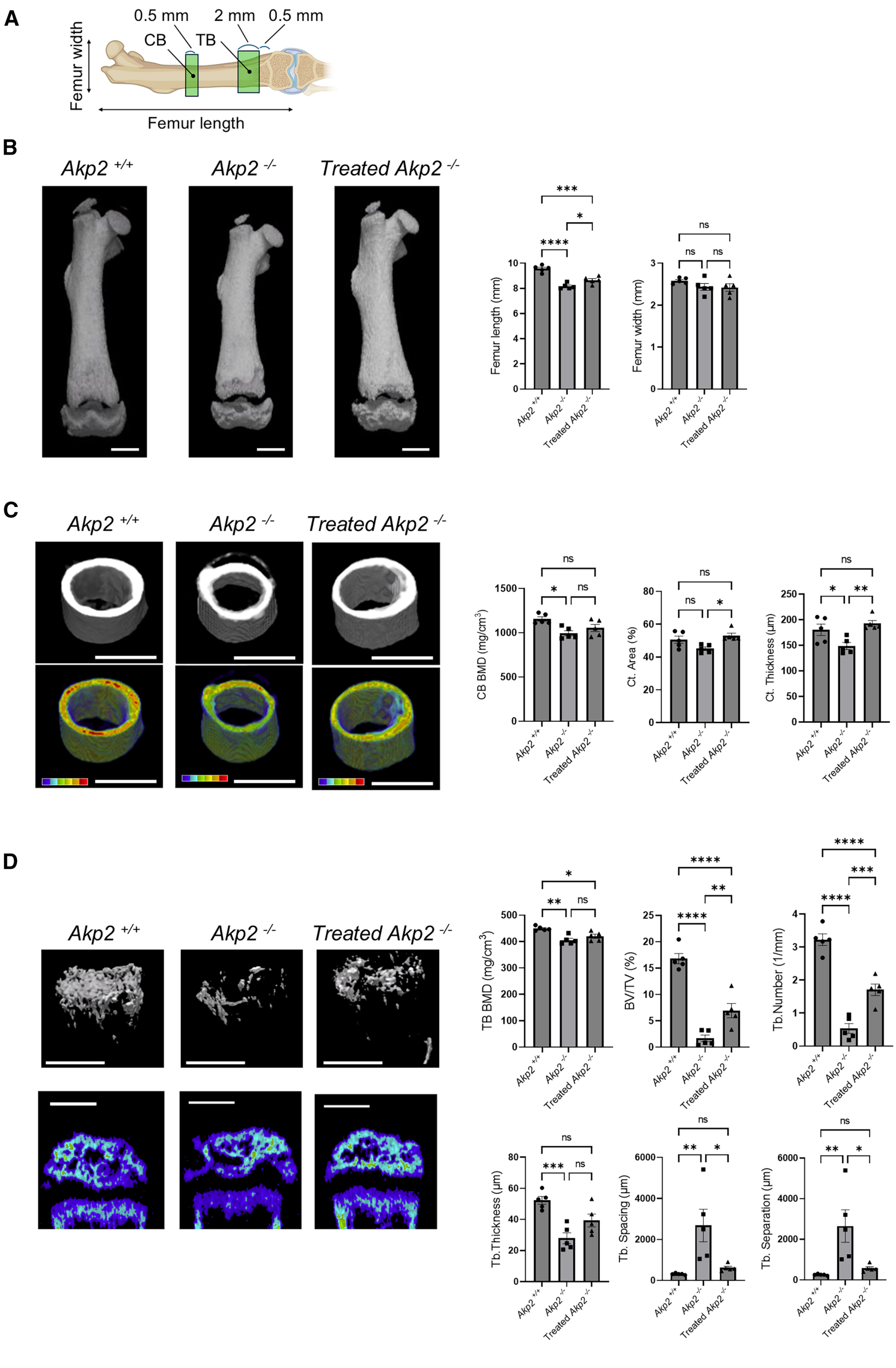

Figure 3. Micro-CT analysis of bone formation following *ex vivo* in-utero cell transplantation in HPP mice

(A) Cortical bone was analyzed with a width of 0.5 mm from the center of the bone, and trabecular bone was analyzed with a width of 2.0 mm from an area of 0.5 mm cephalad to the growth plate (n = 5 in each group).

(B) Treatment significantly increased the length of the femur compared to the untreated *Akp2*$^{-/-}$ group ($p < 0.001$). There was no difference in femur width between the groups ($p = 0.266$).

(legend continued on next page)

the treated $Akp2^{-/-}$ group (TNAP; 132.82 ± 17.49 U/L, $p = 0.013$ and PPi; 10.10 ± 2.82 μM, $p = 0.009$) compared to the untreated $Akp2^{-/-}$ group (Figure 2D). Compared to the untreated $Akp2^{-/-}$ group, the treated $Akp2^{-/-}$ group showed a significant extension in survival rate ($p = 0.005$) (Figure 2E). In the group treated with in-utero cell transplantation (IUT) alone using TNAP-overexpressing human ESC-derived MSCs, no significant increase in TNAP (20.92 ± 1.98 U/L, $p = 0.996$) was observed compared to the untreated $Akp2^{-/-}$ group (Figure S1). Furthermore, wild-type MSCs without gene transfer were administered on gestational day 14.5 and postnatal day 8, but no significant increase in TNAP (35.25 ± 2.39 U/L, $p > 0.999$) was observed compared to the untreated $Akp2^{-/-}$ group (Figure S1). We also confirmed that TNAP promoted bone formation. In the untreated $Akp2^{-/-}$ group, we observed insufficient cartilage tissue formation and malformation of the epiphyseal plate compared to the $Akp2^{+/+}$ group. The treatment led to normal cartilage formation and promoted epiphyseal plate formation in the treated $Akp2^{-/-}$ group (Figure 2F).

Ex vivo in-utero cell transplantation improved bone formation in HPP mice

At 14 days of age, the cortical and trabecular bones were analyzed using micro-computed tomography (CT) images (Figure 3A). The total length of the femur was significantly longer in the treated $Akp2^{-/-}$ group (8.66 ± 0.13 mm) than in the untreated $Akp2^{-/-}$ group (8.17 ± 0.10 mm, $p = 0.030$) (Figure 3B). There was no significant difference in the width of the femur among all groups. The cortical bone ossification area increased with treatment (Figure 3C). In the untreated $Akp2^{-/-}$ group, the cortical bone appears mostly green with low signal intensity, whereas in the treated $Akp2^{-/-}$ group, the cortical bone appeared mostly orange with medium signal intensity, confirming improved calcification. In the treated $Akp2^{-/-}$ group (1053.86 ± 40.81 mg/cm^3; 53.11 ± 1.55%; 192.77 ± 6.03 μm), bone mineral density (BMD), area, and thickness of cortical bone were significantly improved compared to the untreated $Akp2^{-/-}$ group (994.16 ± 29.27 mg/cm^3, $p = 0.420$; 45.14 ± 1.08%, $p = 0.014$; 148.83 ± 6.81 μm, $p = 0.007$) and were enhanced to the same level as the $Akp2^{+/+}$ group (1155.28 ± 25.00 mg/cm^3, $p = 0.109$; 50.62 ± 2.20%, $p = 0.559$; 180.52 ± 11.09 μm, $p = 0.563$). Significant treatment effects were also observed in the trabecular bone analysis in the treated $Akp2^{-/-}$ group (Figure 3D). The images show that the untreated $Akp2^{-/-}$ group had fewer extracted bones, whereas the treated $Akp2^{-/-}$ group had trabecular bone that was evenly formed below the growth plate. Trabecular bone thickness, spacing, and separation in the treated $Akp2^{-/-}$ group (39.34 ± 4.13 μm; 615.58 ± 75.29 μm; 576.24 ± 78.25 μm) were significantly improved compared to the untreated $Akp2^{-/-}$ group (27.92 ± 3.50 μm, $p = 0.088$; 2680.82 ± 795.24 μm, $p = 0.021$; 2652.89 ± 797.83 μm, $p = 0.020$) and were enhanced to the same level as in the $Akp2^{+/+}$ group (52.29 ± 2.52 μm, $p = 0.051$; 313.76 ± 17.41 μm, $p = 0.882$; 261.47 ± 16.26 μm, $p = 0.890$). Trabecular bone volume and number were significantly enhanced in the treated $Akp2^{-/-}$ group (6.94 ± 1.35%, 1.71 ± 0.17/μm) compared to the untreated $Akp2^{-/-}$ group (1.68 ± 0.62%, $p = 0.009$; 0.53 ± 0.15/μm, $p = 0.001$); however, the improvement did not reach the same level as that observed in the $Akp2^{+/+}$ group (16.79 ± 0.97%, $p < 0.001$; 3.23 ± 0.18/μm, $p < 0.001$). The treated $Akp2^{-/-}$ group (420.02 ± 7.56 mg/cm^3) exhibited only a marginal improvement compared with the untreated $Akp2^{-/-}$ group (405.24 ± 7.21 mg/cm^3, $p = 0.272$), and trabecular BMD remained significantly lower than that of the $Akp2^{+/+}$ group (449.30 ± 3.76 mg/cm^3, $p = 0.018$). Trabecular bone mineral content (TB BMC) showed a significant increase in the treated $Akp2^{-/-}$ group (0.066 ± 0.01 mg) compared to the untreated $Akp2^{-/-}$ group (0.013 ± 0.01 mg, $p = 0.012$) (Figure S2).

DISCUSSION

We hypothesized that transplanting TNAP-overexpressing human ESC-derived MSCs into HPP fetuses would provide a continuous source of the deficient enzyme, promoting bone mineralization and improving overall prognosis. This approach was tested in a murine model of HPP, where significant improvements in survival rate, body weight, and bone formation were observed compared to untreated HPP mice. The ESC-derived MSCs generated in this study engrafted at the administration site but showed no evident trafficking to or engraftment within the bone marrow of a recipient mice. Accordingly, we infer that the improvement in bone phenotype was mediated by TNAP overexpressed at the peritoneal site and delivered to the skeleton via the circulation. This mechanism aligns with the current standard of care for HPP, in which exogenously administered TNAP exerts its therapeutic effect by acting directly on the bone matrix following systemic administration. At the same time, TNAP dephosphorylates pyridoxal-5′-phosphate (PLP), the active form of vitamin B6, to produce pyridoxal (PL).[10] PL crosses the blood-brain barrier and is rephosphorylated to PLP. PLP in the brain is involved in the production of various neurotransmitters, so its depletion makes it difficult to maintain normal brain function, often leading to vitamin B6-dependent seizures in severe perinatal HPP. Maintaining normal brain function preserves normal eating habits, and direct promotion of bone formation by TNAP is thought to have led to weight gain in the treatment group.

(C) A micro-CT image of cortical bone and a 3D image of cortical bone mineral density. Treatment significantly improved the cortical area ($p = 0.016$) and cortical thickness ($p = 0.008$) compared to the untreated $Akp2^{-/-}$ group. In the 3D color image, the default settings were used to display low bone density areas in blue and high bone density areas in red within the range of 0–2550 mg/cm^3.

(D) A micro-CT image of trabecular bone and trabecular bone mineral density. Treatment significantly improved bone volume/tissue volume (BV/TV) ($p < 0.001$), trabecular number ($p < 0.001$), trabecular thickness ($p = 0.001$), trabecular spacing ($p = 0.007$), and trabecular separation ($p = 0.007$) compared to the untreated $Akp2^{-/-}$ group. In the 2D color image of the epiphysis, as in Figure 3C, low bone density areas are displayed in blue and high bone density areas in red within the range of 0–2550 mg/cm^3. Statistical significance is shown as *$p < 0.05$, **$p < 0.01$, ***$p < 0.001$, and ****$p < 0.0001$. "ns" means "not significant."

The reason for engraftment without immunosuppressants in the peritoneal cavity is thought to be fetal immune tolerance. It has been suggested that fetal immunity involves immune tolerance mediated by regulatory T cells, which may allow the acceptance of exogenous cells without rejection.[11] Additionally, MSCs possess immune evasion capabilities, making them resistant to attacks from T cells and other immune cells. This is believed to be due to low expression of major histocompatibility complex molecules and the secretion of immune inhibitory factors.[12,13] Therefore, MSCs are considered to be the most suitable cells for transplantation without the use of immunosuppressive agents.

In bone formation, the metabolism of PPi, a potent inhibitor of bone formation, is essential. In this study, TNAP promoted appropriate PPi metabolism, enhancing hydroxyapatite production. Sufficient cartilage tissue formation and the subsequent mineralization process were restored to normal levels. The abnormal arrangement of the growth plate cartilage cell layer was normalized, and various analytical parameters of cortical and trabecular bone in the treated group showed improvement compared to the untreated group. However, unlike other parameters, no significant difference was observed in BMD. BMD is calculated by dividing BMC, the sum of the calcification degrees recognized as trabecular bone in the region of interest (ROI), by the area of trabecular bone. Therefore, in the untreated group, the low degree of trabecular bone calcification, combined with a smaller area of trabecular bone formation, resulted in corrected BMD values within the normal range. In the treated group, the increased degree of bone calcification compared to the untreated group, combined with an increase in the area of trabecular bone formation, led to corrected BMD values that were no longer significantly different from those of the untreated group.

Our study provides compelling evidence for the therapeutic potential of *ex vivo* in-utero gene cell therapy for HPP. By combining the power of gene editing with the regenerative capacity of MSCs,[14,15] we have developed a novel approach to treating this devastating congenital disorder. The perinatal severe form of HPP progresses during fetal development, meaning symptoms are already advanced at birth, necessitating treatment during the fetal period. Therefore, this study specifically focused on phenotypic alterations during the early postnatal period, when the effects of intrauterine cell transplantation are considered most pronounced; however, evaluating therapeutic efficacy and safety beyond this stage remains unfeasible in the current model. To overcome this limitation, we are considering future studies using *Prx1-Cre; Alpl $^{flox/flox}$* mice with longer-term viability to enable long-term evaluation into adolescence and adulthood, including assessments of biomechanical properties, fracture resistance, long-term neurobehavioral and developmental outcomes, and delayed or chronic immune responses. These data will be pivotal for establishing the clinical applicability and comprehensive safety profiles of this therapeutic approach.

The second injection of TNAP-overexpressing ESC-derived MSCs at day 8 effectively increased TNAP levels and improved therapeutic outcomes in our model; however, specific regimens for additional postnatal dosing have not yet been thoroughly investigated. This constitutes a notable methodological limitation, as the protocol for intrauterine transplantation is still in the development stage: the optimal dosing frequency, timing, and total cell number have not been systematically established. However, these findings offer hope for a future in which HPP can be effectively treated before birth, preventing irreversible skeletal deformities that significantly impact patients' quality of life. This platform could also be expanded to other congenital metabolic disorders, offering a new paradigm for in-utero treatment of genetic diseases.[16,17] The development of automated and scalable manufacturing processes for clinical-grade human ESC-derived MSCs will be crucial for the widespread implementation of this therapy.[18] Furthermore, the ethical considerations surrounding in-utero gene therapy must be carefully addressed, ensuring that this powerful technology is used responsibly and for the benefit of patients.

MATERIALS AND METHODS

Ethical statement

Animal experiments were conducted with the approval of the Ethics Committee of the National Center for Child Health and Development (NCCHD; A2003-002), and genetic recombination experiments were performed with the permission of the Genetic Recombination Experiment Safety Committee of the NCCHD (05-2). hESCs used in this study were the SEES-2 human embryonic stem cell line (SEES-2) established at the NCCHD, Japan. All derivations and cultures of hESC lines were conducted in full compliance with the Guidelines on the Derivation and Distribution of Human Embryonic Stem Cells issued by the Ministry of Education, Culture, Sports, Science and Technology (MEXT), Japan (notification no. 156 of 2009), following approval by the Institutional Review Board for hESC research at the NCCHD. The SEES-2 is officially registered in the Japanese national hESC registry and has been previously reported in peer-reviewed publications (Akutsu H et al. Regen Ther 2015).

Characterization of human ESC-derived MSCs

As previously reported, we have produced human ESC-derived MSCs.[14] The cells were cultured in 500 mL of Minimum Essential Medium α (αMEM) supplemented with 50 mL of fetal bovine serum (FBS) and 5 mL of penicillin/streptomycin at 37°C in a 5% CO2 atmosphere and passaged every 7 days using trypsin. The cells were evaluated for cell surface antigens using fluorescence-activated cell sorting. A total of 1.0×10^6 human ESC-derived MSCs were diluted in 1 mL phosphate-buffered saline (PBS), and after dead cells were fluorescently stained (Zombie Violet Fixable Viability Kit), they were incubated with each antibody. Antibodies against surface antigens were used according to the guidelines of the International Society for Cell and Gene Therapy, including CD105, CD73, CD90, CD45, CD34, CD14, CD19, and HLA-DR (Milteyi Biotec). The fluorescence intensity of the cells was measured using the SONY CELL Sorter SH800. Analysis was performed using the SONY cell sorter Software. The StemPro Differentiation Kit (Gibco, #A1007001, #A1007101, #A1007201) was used as the culture medium for

differentiation induction. For osteogenesis and adipogenesis, 4.0×10^4 or 2.0×10^4 cells were seeded in a 12-well plate. For cartilage differentiation, 1.6×10^7 cells/ml were cultured at 5 μL/well in a 96-well plate. The culture medium was changed every 3 days, and histopathological staining was performed on day 21.

TNAP-overexpressing human ESC-derived MSCs

The *hALPL-D10* gene was inserted using a lentiviral vector. The *hALPL-D10* gene and the *ZsGreen* gene were designed to be joined by the internal ribosome entry site (IRES) sequence and co-expressed. We employed the EF1α promoter to drive *hALPL-D10* expression. Vesicular stomatitis virus G protein (VSV-G) was used to pseudotype the lentiviral vector (Figure S3). Human ESC-derived MSCs were seeded in culture medium and transduced with a lentivirus vector at MOI of 20. Polybrene (6 μg/mL) was added immediately before administration. After 20 h, the virus was removed, and 2 days later, TNAP activity in the supernatant and the amount of the *hALPL-D10* gene in the cells were measured. TNAP was reacted with *p*-nitrophenyl phosphate (FUJIFILM, #149–02342) for 30 min, and the *p*-nitrophenol produced was evaluated using a colorimetric assay at 405 nm (PerkinElmer, 2030 Multilabel Reader). One unit was defined as the amount of enzyme that can metabolize 1 μmol of *p*-nitrophenyl phosphate in 1 min.

For analysis of *hALPL-D10* gene expression, RNA was extracted from the cells in the dish using ISOGEN (NIPPON GENE, # 311–02501). cDNA was synthesized using SuperScript IV VILO Master Mix (Invitrogen, #11756050). Using the completed cDNA as a template, we quantified the amplified PCR products using Platinum SYBR Green qPCR SuperMix-UDG with ROX (Invitrogen, #11733046) on the Applied Biosystems QuantStudio 12 K Flex Real-Time PCR System. The primers for the *hALPL-D10* gene region were as follows: forward 5′-GGAACTCCTGACCCTTGACC-3′ and reverse 5′-CCAGCAAGAAGAAGCCTTTG-3’. We selected the glyceraldehyde-3-phosphate dehydrogenase gene as the housekeeping gene and used the following primers: forward, 5′ TGTTGCCATCAATGACCCCTT 3’ and reverse, 5′ CTCCACGA CGTACTCAGCG 3’.

In-utero cell transplantation

Akp2 mice were used as an HPP model. *Akp2* mice are deficient in TNAP due to a genetic alteration in exon 6 of the *TNAP* gene, and $Akp2^{-/-}$ mice develop a severe form of HPP in the perinatal period. All $Akp2^{-/-}$ mice were raised on a 325 ppm/10 kg pyridoxine-containing diet. DNA was extracted from toe tips using the QIAamp DNA Mini kit (QIAGEN, #51306) to assess the genotype of the mice. DNA was amplified using a BIO-RAD C1000 Touch Thermal Cycler, and the PCR products were electrophoresed. The primers used were as follows: forward 5′-AGTCCGTGGGCATTGTG ACTA-3′ and reverse 5′-TGCTGCTCCACTCACGTCGAT-3’.

On day 14.5 of gestation, the mice were anesthetized with isoflurane, and their uterui were exposed by laparotomy under a microscope. TNAP overexpressing ESC-derived MSCs (1.0×10^6 cells) were suspended in 5 μL of PBS and administered intraperitoneally into the abdominal cavity of each fetus using a 33G needle. Following the procedure, the abdomen was closed, and carprofen was administered subcutaneously as an analgesic. Genotyping was performed immediately after birth, and in the treated group, only $Akp2^{-/-}$ mice were selected, while in the untreated group, $Akp2^{+/+}$ and $Akp2^{-/-}$ mice were selected. An additional dose of 1.0×10^7 cells per fetus was administered intraperitoneally on day 8 after birth in the treated group. The mice were weighed on days 0, 7, and 14 after birth, and blood was collected from the inferior vena cava on day 14 after birth. The blood was centrifuged at 12,000 rpm for 5 min, and the TNAP and PPi activity were evaluated in the supernatant.

Micro-CT analysis

Harvested femurs were stored and fixed in 4% paraformaldehyde (PFA) overnight at 4°C. The fixed femurs were scanned using the CosmoScan FX (Summit Pharmaceuticals International Corporation, Tokyo, Japan) with standard settings. Imaging conditions were as follows: tube voltage, 90 kV; tube current, 88 μA; voxel size, 20 × 20 × 20 μm. The voxel size was then changed to 10 × 10 × 10 μm for more detailed cancellous bone analysis, and only the area near the growth plate was rescanned. The image data were constructed using TRI/3D-SRF software (Ratoc System Engineering, Tokyo, Japan; n = 5). The default settings were used to display low bone density areas in blue and high bone density areas in red within the range of 0–2550 mg/cm^3. Trabecular bone analysis was performed with an ROI of 2 mm thickness above a location 0.5 mm from the growth plate to avoid primary trabecular bone. The following trabecular bone analysis parameters were measured using TRI/3D-SRF: trabecular BMD (TB BMD), bone volume/tissue volume (BV/TV), trabecular thickness (Tb.Th), trabecular number (Tb.N), trabecular separation (Tb.Sp), trabecular spacing (Tb.Spac) (n = 5). For cortical bone analysis, an ROI of 1 mm in width was set at the center of the bone shaft. Similarly, the following parameters were measured: cortical BMD (CB BMD), average cortical thickness (Ct.Th), and cortical area fraction (Ct.Ar) (n = 5).

Statistical analysis

The paired t test was used to measure *hALPL-D10* gene expression and TNAP activity. One-way ANOVA was used to analyze mouse body weight, serum TNAP and PPi activity, and micro-CT analysis. The log-rank test was used to analyze survival rates. All measurements are presented as mean ± SEM. Statistical analyses were conducted in GraphPad Prism 9.5.1 (GraphPad Software).

DATA AND CODE AVAILABILITY

The datasets analyzed during this study are available from the corresponding author upon reasonable request.

ACKNOWLEDGMENTS

We thank M. Ichinose for expert technical assistance and E. Suzuki and K. Saito for secretarial work. This research was supported by the Acceleration Program of R&D and Implementation for Regenerative Medicine and Cell and Gene Therapy from the Japan Agency for Medical Research and Development (AMED) under grant no.

JP24bm1323001, JSPS KAKENHI under grant no. JP22K15911, and the Jikei University Research Fund for Graduate Students.

AUTHOR CONTRIBUTIONS

N.K., A.H., and A.U. designed the research, performed the experiments, analyzed the data, and wrote the manuscript. A.T.-N., M.K., H.A., H.S., O.S., and A.O. supervised the study. All authors reviewed and edited the manuscript.

DECLARATION OF INTERESTS

The authors declare no competing interests.

DECLARATION OF GENERATIVE AI AND AI-ASSISTED TECHNOLOGIES IN THE WRITING PROCESS

While preparing this work, N.K. and A.U. utilized DeepL, Grammarly, and ChatGPT to check English grammar. After using these tools, N.K. and A.U. carefully reviewed and edited the content as necessary and took full responsibility for the final content of the publication.

SUPPLEMENTAL INFORMATION

Supplemental information can be found online at https://doi.org/10.1016/j.omta.2026.201696.

REFERENCES

1. Weiss, M.J., Cole, D.E., Ray, K., Whyte, M.P., Lafferty, M.A., Mulivor, R.A., and Harris, H. (1988). A missense mutation in the human liver/bone/kidney alkaline phosphatase gene causing a lethal form of hypophosphatasia. Proc. Natl. Acad. Sci. USA *85*, 7666–7669.
2. Millán, J.L., and Plotkin, H. (2012). Hypophosphatasia - pathophysiology and treatment. Actual. Osteol. *8*, 164–182.
3. Mornet, E., Yvard, A., Taillandier, A., Fauvert, D., and Simon-Bouy, B. (2011). A molecular-based estimation of the prevalence of hypophosphatasia in the European population: Prevalence of hypophosphatasia. Ann. Hum. Genet. *75*, 439–445.
4. Watanabe, A., Karasugi, T., Sawai, H., Naing, B.T., Ikegawa, S., Orimo, H., and Shimada, T. (2011). Prevalence of c.1559delT in ALPL, a common mutation resulting in the perinatal (lethal) form of hypophosphatasia in Japanese and effects of the mutation on heterozygous carriers. J. Hum. Genet. *56*, 166–168.
5. Ozono, K., and Michigami, T. (2011). Hypophosphatasia now draws more attention of both clinicians and researchers: a commentary on Prevalence of c. 1559delT in ALPL, a common mutation resulting in the perinatal (lethal) form of hypophosphatasias in Japanese and effects of the mutation on heterozygous carriers. J. Hum. Genet. *56*, 174–176.
6. Leung, E.C.W., Mhanni, A.A., Reed, M., Whyte, M.P., Landy, H., and Greenberg, C.R. (2013). Outcome of perinatal hypophosphatasia in manitoba mennonites: a retrospective cohort analysis. JIMD Rep. *11*, 73–78.
7. Offiah, A.C., Vockley, J., Munns, C.F., and Murotsuki, J. (2019). Differential diagnosis of perinatal hypophosphatasia: radiologic perspectives. Pediatr. Radiol. *49*, 3–22.
8. Drury, S., Williams, H., Trump, N., Boustred, C., Chitty, L.S., GOSGene, Lench, N., and Scott, R.H. (2015). Exome sequencing for prenatal diagnosis of fetuses with sonographic abnormalities. Prenat. Diagn. *35*, 1010–1017.
9. Whyte, M.P., Simmons, J.H., Moseley, S., Fujita, K.P., Bishop, N., Salman, N.J., Taylor, J., Phillips, D., McGinn, M., and McAlister, W.H. (2019). Asfotase alfa for infants and young children with hypophosphatasia: 7 year outcomes of a single-arm, open-label, phase 2 extension trial. Lancet Diabetes Endocrinol. *7*, 93–105.
10. Narisawa, S., Wennberg, C., and Millán, J.L. (2001). Abnormal vitamin B6 metabolism in alkaline phosphatase knock-out mice causes multiple abnormalities, but not the impaired bone mineralization. J. Pathol. *193*, 125–133.
11. Hirayama, M., Azuma, E., and Komada, Y. (2012). Tolerogenic effect of non-inherited maternal antigens in hematopoietic stem cell transplantation. Front. Immunol. *3*, 135.
12. Oh, J.Y., Kim, H., Lee, H.J., Lee, K., Barreda, H., Kim, H.J., Shin, E., Bae, E.-H., Kaur, G., Zhang, Y., et al. (2022). MHC Class I Enables MSCs to Evade NK-Cell-Mediated Cytotoxicity and Exert Immunosuppressive Activity. Stem Cell. *40*, 870–882.
13. Gao, F., Chiu, S.M., Motan, D.A.L., Zhang, Z., Chen, L., Ji, H.-L., Tse, H.-F., Fu, Q.-L., and Lian, Q. (2016). Mesenchymal stem cells and immunomodulation: current status and future prospects. Cell Death Dis. *7*, e2062.
14. Ando, Y., Saito, M., Machida, M., Yoshida-Noro, C., Akutsu, H., Takahashi, M., Toyoda, M., and Umezawa, A. (2017). Can Human Embryonic Stem Cell-Derived Stromal Cells Serve a Starting Material for Myoblasts? Stem Cells Int. *2017*, 7541734.
15. Chen, J., Horiuchi, S., Kuramochi, S., Kawasaki, T., Kawasumi, H., Akiyama, S., Arai, T., Morinaga, K., Kimura, T., Kiyono, T., et al. (2024). Human intestinal organoid-derived PDGFRα + mesenchymal stroma enables proliferation and maintenance of LGR4 + epithelial stem cells. Stem Cell Res. Ther. *15*, 16.
16. Botor, M., Fus-Kujawa, A., Uroczynska, M., Stepien, K.L., Galicka, A., Gawron, K., and Sieron, A.L. (2021). Osteogenesis Imperfecta: Current and Prospective Therapies. Biomolecules *11*, 1493. https://doi.org/10.3390/biom11101493.
17. Ihara, N., Akihiro, U., Onami, N., Tsumura, H., Inoue, E., Hayashi, S., Sago, H., and Mizutani, S. (2015). Partial rescue of mucopolysaccharidosis type VII mice with a lifelong engraftment of allogeneic stem cells in utero. Congenit. Anom. *55*, 55–64.
18. Umezawa, A., Fukuda, A., Horikawa, R., Uchida, H., Enosawa, S., Oishi, Y., Nakamura, N., Sasaki, K., Yanagi, Y., Shimizu, S., et al. (2025). First-in-human clinical study of an embryonic stem cell product for urea cycle disorders. Stem Cell Res. Ther. *16*, 120.

OMTA, Volume *34*

Supplemental information

In-utero cell transplantation for hypophosphatasia with gene-edited hESC-derived MSCs in a murine model

Naoya Kitamura, Akihiro Hasegawa, Aki Takahashi-Nakamura, Masataka Kasahara, Hidenori Akutsu, Haruhiko Sago, Osamu Samura, Aikou Okamoto, and Akihiro Umezawa

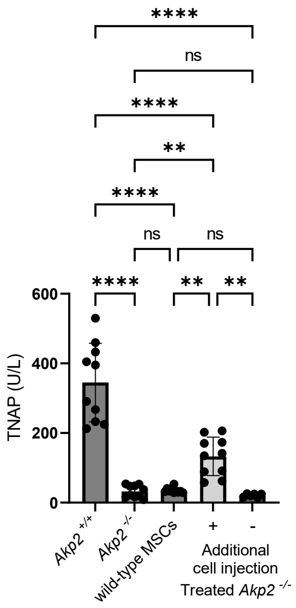

Figure S1. Serum TNAP levels at 14 days postnatally.

Akp2 $^{+/+}$: Wild-type *Akp2* mice. *Akp2* $^{-/-}$: Untreated HPP mice. Wild-type MSCs: Mice administered MSCs without gene transfer at E14.5 and postnatal day 8. Additional cell injection Treated *Akp2* $^{-/-}$: Groups receiving (+) or not receiving (–) an additional injection at postnatal day 8 after receiving TNAP-overexpressing human ESC-derived MSCs at E14.5.

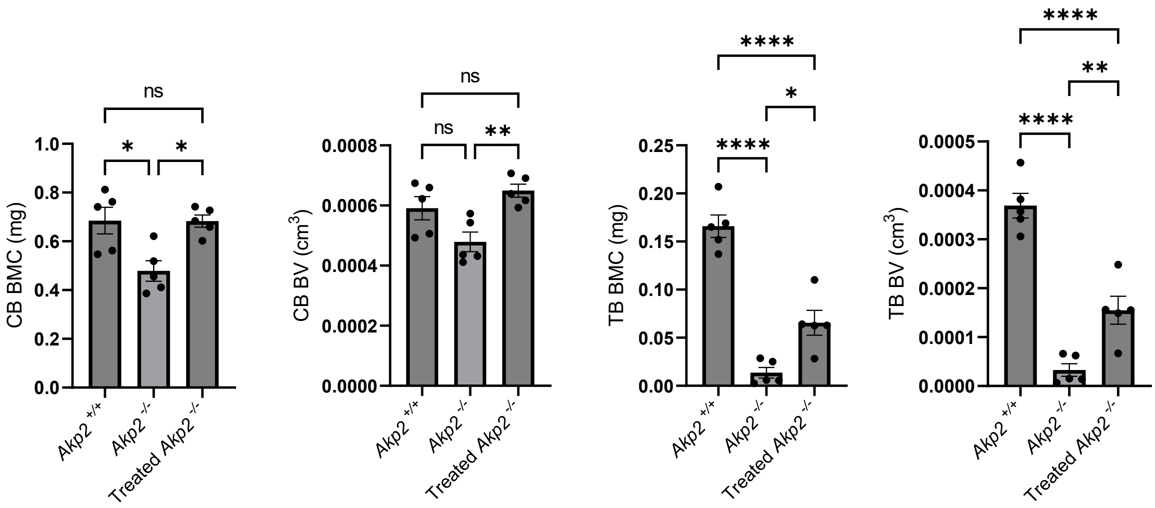

Figure S2. Additional micro-CT analysis data of bone formation 14 days postnatally. BMC represents the total calcification level recognized as bone within the ROI, and BV indicates bone volume. Data are shown for cortical (CB) and trabecular (TB) bone, respectively. The treatment resulted in a significant improvement in both parameters compared with the untreated *Akp2* $^{-/-}$ group.

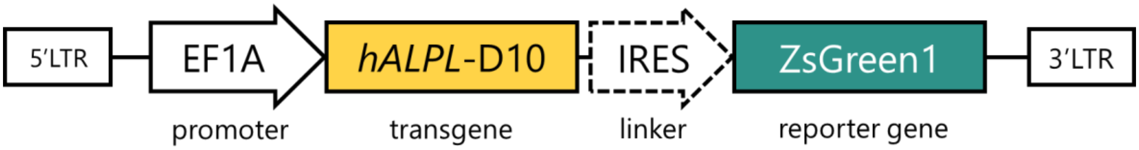

Figure S3. Schematic map of the lentiviral expression vector used to transduce MSCs with the hALPL-D10 and ZsGreen genes.
The two genes are co-expressed from a single bicistronic transcript via an IRES sequence.
